# Epidemiology and spatiotemporal analysis of acute hemorrhagic conjunctivitis in Zhejiang province, China (2004–2023)

**DOI:** 10.3389/fpubh.2025.1509495

**Published:** 2025-02-03

**Authors:** Wanwan Sun, Yijuan Chen, Shuwen Qin, Ziping Miao

**Affiliations:** Department of Infectious Diseases, Zhejiang Provincial Center for Disease Control and Prevention, Hangzhou, China

**Keywords:** acute hemorrhagic conjunctivitis, spatiotemporal analysis, epidemiology, clustering, outbreak

## Abstract

**Background:**

Acute hemorrhagic conjunctivitis (AHC) has become a significant public health issue in Zhejiang province. However, the analysis of epidemiological characteristics and spatio-temporal patterns of AHC in Zhejiang province has not been studied yet.

**Methods:**

Monthly cases of AHC from 2004 to 2023 reported at the county level in Zhejiang province were obtained from the China Information System for Disease Control and Prevention. Demographic features, yearly county incidence, global spatial autocorrelation, local spatial autocorrelation analyses, and temporal and space–time cluster analysis were performed to identify and visualize the spatiotemporal patterns of AHC cases in Zhejiang province. The epidemiological characteristics of AHC outbreaks in the same period in Zhejiang province were also compared.

**Results:**

A total of 52,119 AHC cases were reported in Zhejiang province, yielding an average incidence rate of 5.37 per 100,000. No fatalities were reported. The average age of those affected was 25.44 ± 4.37 years, with the highest incidence (16.70%) among individuals aged between 10 and 19 years. Seasonal peaks occur from August to October each year. Students and farmers experienced the highest incidence rates of infection. Significant positive spatial correlations for AHC were observed in Zhejiang province in the years 2007 (Moran’s I = 0.095, *p* = 0.039), 2009 (Moran’s I = 0.075, *p* = 0.031), and 2011 (Moran’s I = 0.173, *p* = 0.034), indicating spatial clustering. Spatiotemporal scanning identified two distinct clusters: Cluster 1 and Cluster 2. Cluster 1, characterized by a relative risk of 21.44 (*p* < 0.001), was located in northeastern Zhejiang province, comprising 30 counties, with an active period from 1st September 2010 to 30th September 2010. Compared to low-risk regions, high-risk counties exhibited a different demographic profile with a higher proportion of men, older people, and farmers. Among the affected students during outbreaks, the predominant symptoms were conjunctival congestion, increased eye secretions, eye swelling, eye pain, photophobia and tearing, while the incidence of fever was relatively low.

**Conclusion:**

The results of this study demonstrate the spatiotemporal heterogeneity of AHC cases in Zhejiang province and underscore the necessity for targeted prevention and control measures in high-risk areas to mitigate transmission and occurrence.

## Introduction

The pathogens responsible for acute hemorrhagic conjunctivitis (AHC) are diverse, mainly microribonucleic acid viruses, with enterovirus type 70 (EV70) being the most prevalent, followed by Coxsackie virus A group 24 variant (CA24v) and variants of adenovirus types 3, 7, 8, 11, 19, and 37 (1, 2). Patients infected with AHC mainly exhibit symptoms such as eye redness, stinging, increased eye secretion, tearing, and watery discharge (3). AHC is a self-limited eye infectious disease of the eye, with an incubation period generally ranging from 12 h to 48 h and up to a maximum of 6 days (4).

Patients are the main source of infection, with the main mode of transmission being contact transmission through eye-to-hand, object, and water-to-eye contact. Additionally, the virus can be transmitted via the fecal-oral route through contaminated water (5). AHC is noted for its rapid onset and high infectivity. Children aged 0–9 years are most commonly affected (6), often leading to global pandemics and frequent outbreaks in schools (7). Relevant reports indicated that the inflammatory response of conjunctivitis can spread to the cornea and cause a certain degree of visual impairment that may last over months or even years (8).

Since the first occurrence of AHC in Ghana in 1969 (9), it has spread rapidly worldwide. In recent decades, more than 10 million AHC cases have been reported worldwide (10), including several large outbreaks in Asia and South America. The largest outbreak of AHC was reported in South Korea in 2002, with over 1 million cases (11). In 2014, Thailand had an outbreak of AHC, with 300,000 cases over 3 months (12). The first outbreak of AHC in China was reported in Hong Kong in 1971 (13), and then spread to all provinces and cities. In 2022, influenza, other infectious diarrhoeal, hand-foot-mouth disease, epidemic mumps, and acute hepatitis C will be listed as the top five incidence rates of Class C infectious diseases in China (14). The incidence of AHC varies greatly in different regions in mainland China. Guangxi, Guangdong, Anhui, and Hubei are provinces with a larger number of cases from 2013 to 2020 (6). Currently, AHC is still an important public health issue in China, and its’ epidemic feature is worthy of further study.

In recent years, geographic information systems and spatial statistics have been widely applied to study the distribution characteristics of infectious diseases (15, 16). However, there is limited research on the spatial epidemiological characteristics of AHC across a province. This study analyzed the spatiotemporal aggregation characteristics of AHC incidence in Zhejiang province in the past 20 years (from 2004 to 2023). The results of the epidemiologic and spatiotemporal analysis in this article help provide a basis for targeting public health measures and allocation of health resources in disease prevention and control.

## Materials and methods

### Study area

Zhejiang Province is located on the southeast coast of China. Zhejiang province has 11 prefecture-level cities, with 90 municipal or county-level cities (including one autonomous county) (17). The epidemiological and spatiotemporal analyses in this study were carried out on the basis of counties.

### Data collection

The data on AHC cases and outbreaks in Zhejiang province from January 2004 to December 2023 were obtained from the China Information System for Disease Control and Prevention (CISDCP) and the Management Information System for Public Health Emergencies, respectively. The information included the age, gender, occupation, residential address, date of illness onset, and date of illness diagnosis for the AHC cases.

The cases’ communication information, address, and other personal information were not involved in this study. The diagnosis of AHC refers to the health industry standards for the diagnosis of AHC issued by the National Health Commission of the People’s Republic of China (WS 217–2008; WS 217–2021) (18). See Supplementary material. The demographical data of Zhejiang province was downloaded from the Zhejiang Statistical Yearbook (https://tjj.zj.gov.cn/col/col1525563/index.html). Maps of China and Zhejiang province were obtained from the National Basic Geographic Information System.

### Spatial autocorrelation analysis

Spatial autocorrelation analysis is a spatial method used to quantify the spatial autocorrelation association based on the location of cases in different study regions. This analysis is typically divided into “global” and “local” categories. Both global and local Moran’s I indices were employed to determine the presence of significant spatial autocorrelation regions for AHC within the study area. Moran’s I coefficients, which range from −1 to 1, were calculated using a Z-test. Values approaching 1 indicate a more significant aggregation of cases, while those approaching −1 suggest a more dispersed distribution. A Moran’s I value near 0 indicates a global random distribution. Z and *p* values were used to evaluate the significance of Moran’s I coefficients (19).

### Local spatial autocorrelation analysis

In this process, Moran’s I for local indication of spatial autocorrelation (LISA) measures the similarity or difference between the incidence of the given county unit and those of surrounding counties. LISA was adopted to identify significant spots into four categories: hotspots (high-high), cold spots (low-low), and outliers (high-low, low-high) of AHC. The significance level of aggregations was determined by the Z score generated by comparison of the local Moran’s I data for the average incidence in each county. A high positive Z score represented that the surroundings had spatial clusters (high-high: high-value spatial clusters or low-low: low-value spatial clusters), and a low negative Z score represented the presence of spatial outliers (high-low: high values surrounded with low values or low-high: low values surrounded with high values) (20, 21).

### Spatiotemporal cluster analysis

Spatiotemporal aggregation analysis was used to detect the location of possible high-risk space–time clusters for monthly AHC cases from 2004 to 2023 at the county level.

The basic principle is that Kulldorff’s space–time scan statistic (SaTScan software) scanned the clusters by the variable-sized circular scanning window around the centroid of each county group (22). The null hypothesis assumed that the incidence’s relative risk (RR) was the same within the window compared to outside. The difference in the number of cases inside and outside the scanning window was tested by log-likelihood ratio (LLR). The scanning window with the largest statistically significant LLR value is defined as the primary cluster, and other windows with significant LLR values are considered secondary clusters (23). A statistical test was evaluated using the Monte Carlo hypothesis simulation (set to the default value of 999) to calculate the corresponding *p*-value. The larger the LLR value (*p* < 0.05), the greater the probability that the area contained in the dynamic window is an aggregation area (24). In this study, according to the county-level AHC cases, demographic data, and geographical data, the maximum spatial cluster size was set to 50% (25) of the population at risk in the spatial window in the process to fit discrete Poisson models (16). The scanning time extended from 1 January 2004 to 31 December 2023, with the time interval set to ‘Month’ to identify the aggregation of spatiotemporal clusters throughout the study period.

### Statistical software

In this study, the R software program (v.4.3.1, https://www.R-project.org/) was used in descriptive statistical analysis, including investigating the population distribution, occupational composition, seasonal characteristics, diagnosis features, and outbreak investigation of AHC cases. GeoDa software (version 1.22, Spatial Analysis Laboratory, Urbana, IL, USA) was used for the global and local spatial autocorrelation analysis. The spatiotemporal clusters were detected by SaTScan software (version 10.1.3 Martin Kulldorff, National Cancer Institute, Bethesda, MD, USA). ArcGIS software (version 10.3 ESRI, Redlands, CA, USA) was used for mapping and visualization analysis. All results were considered statistically significant when the *p* < 0.05 for both sides.

## Results

### Epidemiological characteristics

**Figure 1:**
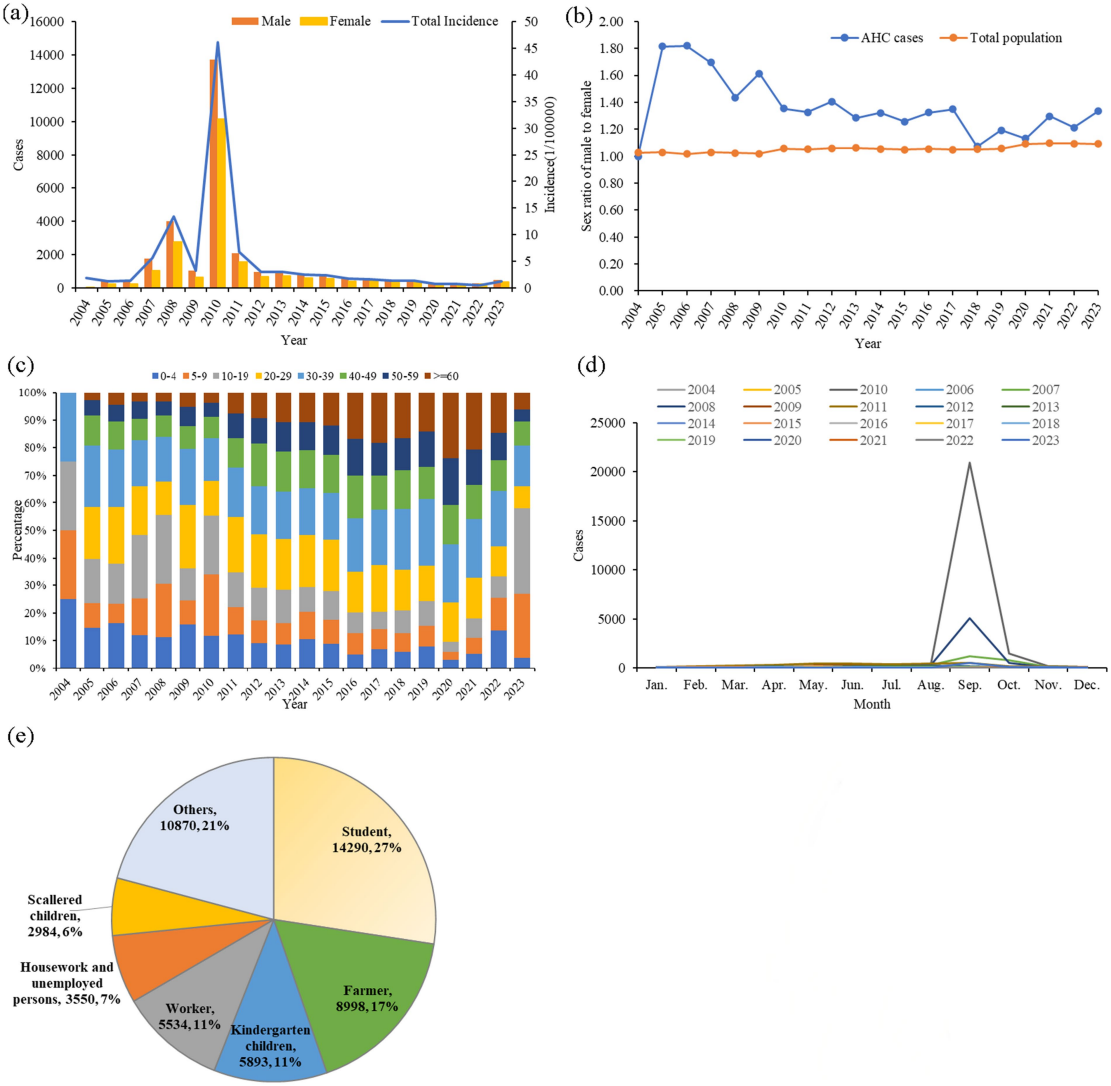
Epidemiological characteristics of AHC in Zhejiang province from 2004 to 2023. **(A)** Annual case numbers of AHC among men and women, along with total annual incidence; **(B)** annual male-to-female ratios in AHC cases and compared to the total population in Zhejiang province; **(C)** age distribution of annual AHC cases; **(D)** monthly distribution of AHC cases; and **(E)** occupational distribution among AHC cases.

From 2004 to 2023, a total of 52,119 AHC cases were reported in Zhejiang province, with an annual reported incidence of 5.37 per 100,000 (ranging from 0.77 per 100,000 in 2022 to 46.17 per 100,000 in 2010). The highest incidence was observed in 2010 (46.17 per 100,000), with the seasonal peak in September in almost all years (Figures 1A,D). During this study period, there were 30,212 male cases and 21,912 female cases. No fatalities were reported in 20 years. The average gender ratio of male to female is 1.38:1, which is higher than the gender ratio of common residents in Zhejiang Province each year (other than the year 2004) (Figure 1B).

The average age of all study cases was 25.44 ± 4.37 years. The largest age group was 10–19 years old, comprising 18.33% of cases, followed by those aged 30–39 years (16.78%) and 5–9 years (16.70%), as illustrated in Figure 1C. The most common occupations among AHC cases were students (14,290 cases, 27.42%), followed by individuals in other occupations (10,870 cases, 20.85%), farmers (8,998 cases, 17.26%), kindergarten children (5,893 cases, 11.31%), and workers (5,534 cases, 10.62%), as shown in Figure 1E. All cases in this study were clinically diagnosed.

### Incidence map

**Figure 2:**
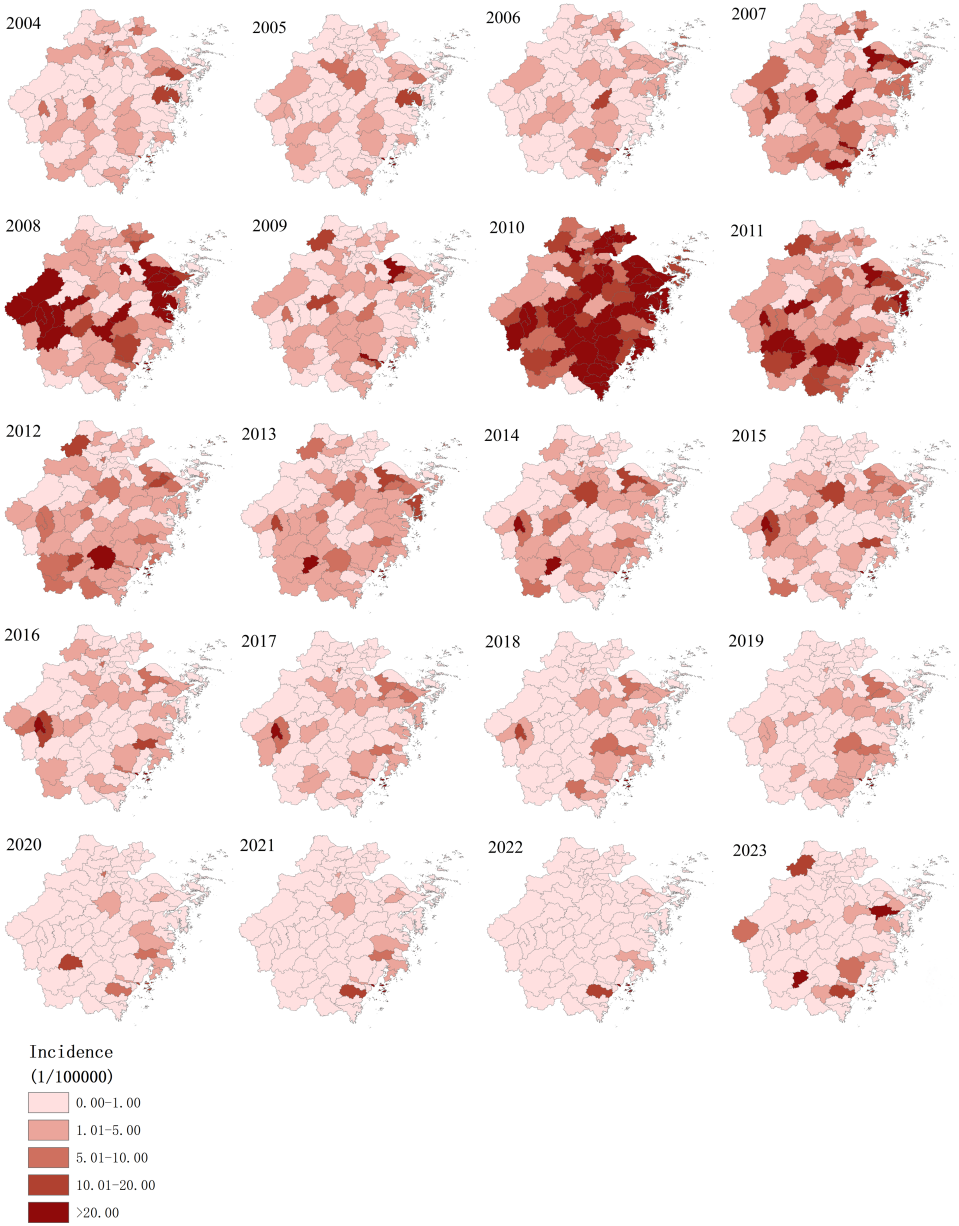
Annual incidence maps of AHC in Zhejiang province from 2004 to 2023.

The annualized incidence rate of AHC in Zhejiang province was mapped at the county level, ranging from 0.00 to 654.49 per 100,000 people. The high-incidence counties of AHC have changed over the years. The top 10 counties in terms of annual incidence were Dongtou (654.49 per 100,000), Pingyang (289.89 per 100,000), Yuyao (275.32 per 100,000), Longwan (156.02 per 100,000), Ninghai (142.37 per 100,0001), Lucheng (132.64 per 100,000), Yongjia (117.89 per 100,000), Tongxiang (110.80 per 100,0001), Wucheng (103.82 per 100,000) and Sanmen (101.95 per 100,0001), a majority of the top 10 counties are located in the southeast of Zhejiang Province (Figure 2).

### Spatial autocorrelation analyses

**Figure 3:**
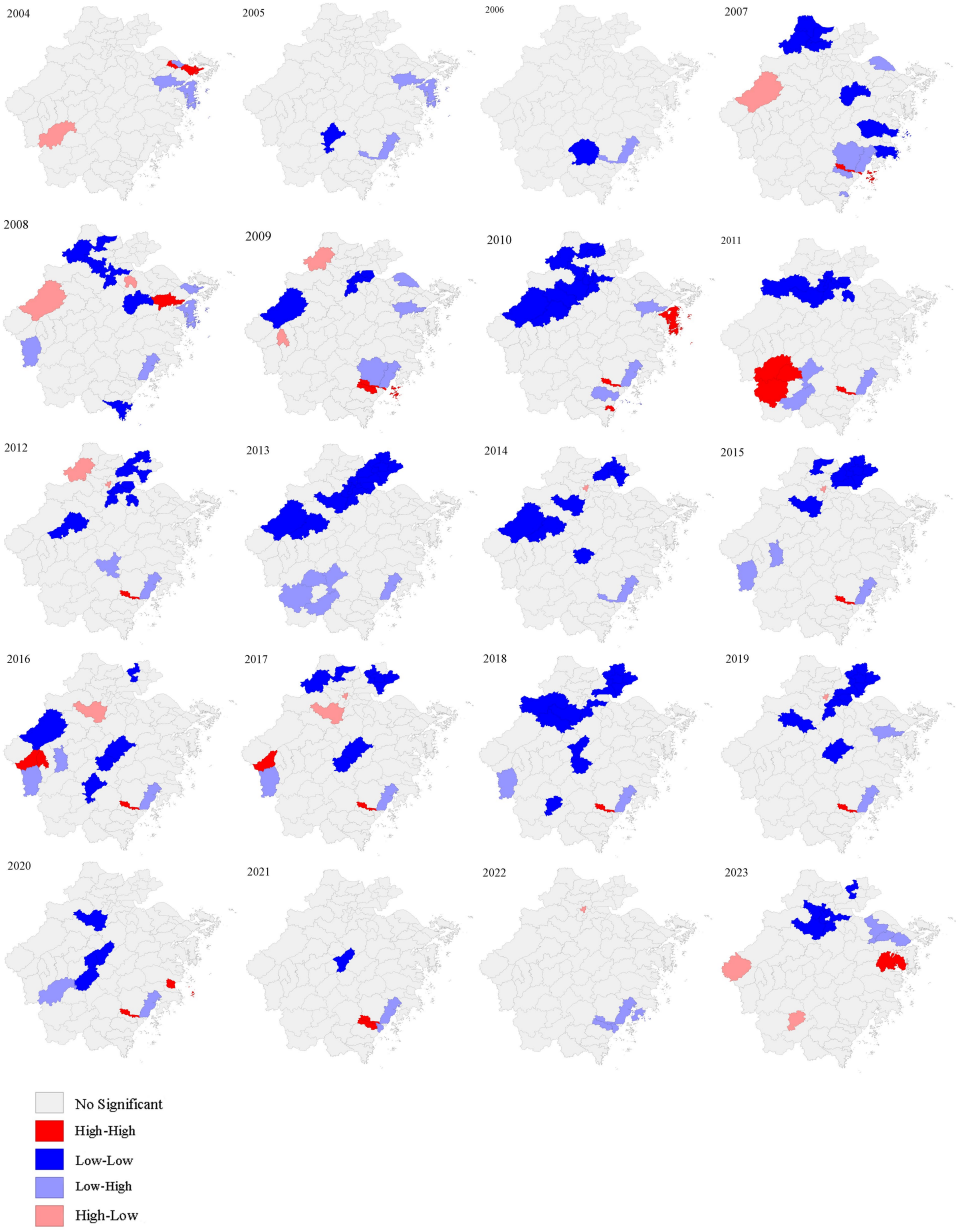
Annual local spatial autocorrelation maps of AHC in Zhejiang province from 2004 to 2023.

Except for 2007 (Moran’s I = 0.095, *p* = 0.039), 2009 (Moran’s I = 0.075, *p* = 0.031), and 2011 (Moran’s I = 0.173, *p* = 0.034), the global spatial autocorrelation analysis of AHC incidence in other years did not show significant global correlation (Table 1). LISA analysis was performed to identify significant hotspots (High-High), cold spots (Low-Low), and outliers (High-Low and Low-High) of AHC cases in Zhejiang province from 2004 to 2023 (Figure 3). A total of 33 hotspots, 131 cold spots, and 82 outliers (63 low-high and 19 high-low clusters) were detected from 2004 to 2023. Hotspots were observed in southern counties such as Lucheng, Ouhai, and Longwan counties, and the eastern counties such as Zhenhai, Beilun, Fenghua, Xiangshan, and Ninghai counties. Years of 2005–2006 and 2013–2014 had detected no hotspot. Notably, Lucheng County was found to be a high-high cluster 12 times during the study period (Figure 3).

**Table 1:** The global spatial autocorrelation of AHC in Zhejiang province from 2004 to 2023.

Year	Moran’s I	Z-Score	*P*-Value	Mean	SD
2004	−0.041	−0.343	0.407	−0.013	0.080
2005	−0.049	−0.749	0.179	−0.010	0.052
2006	−0.013	−0.123	0.451	−0.011	0.019
2007	0.095	2.255	0.039	−0.010	0.047
2008	0.049	0.901	0.161	−0.011	0.067
2009	0.075	2.898	0.031	−0.012	0.030
2010	0.122	1.818	0.052	−0.009	0.072
2011	0.173	2.388	0.034	−0.010	0.076
2012	0.038	0.915	0.178	−0.010	0.053
2013	0.003	0.126	0.261	−0.006	0.071
2014	−0.003	0.173	0.296	−0.009	0.033
2015	0.010	0.460	0.182	−0.010	0.044
2016	0.070	1.124	0.075	−0.009	0.071
2017	0.080	1.240	0.056	−0.010	0.072
2018	0.029	0.832	0.149	−0.010	0.046
2019	0.039	1.891	0.056	−0.011	0.027
2020	0.029	0.598	0.160	−0.013	0.070
2021	0.040	0.871	0.102	−0.010	0.058
2022	−0.012	−0.040	0.318	−0.010	0.032
2023	−0.034	−0.325	0.500	−0.011	0.071

SD, standard deviation.

### Temporal and space–time cluster analysis

**Figure 4:**
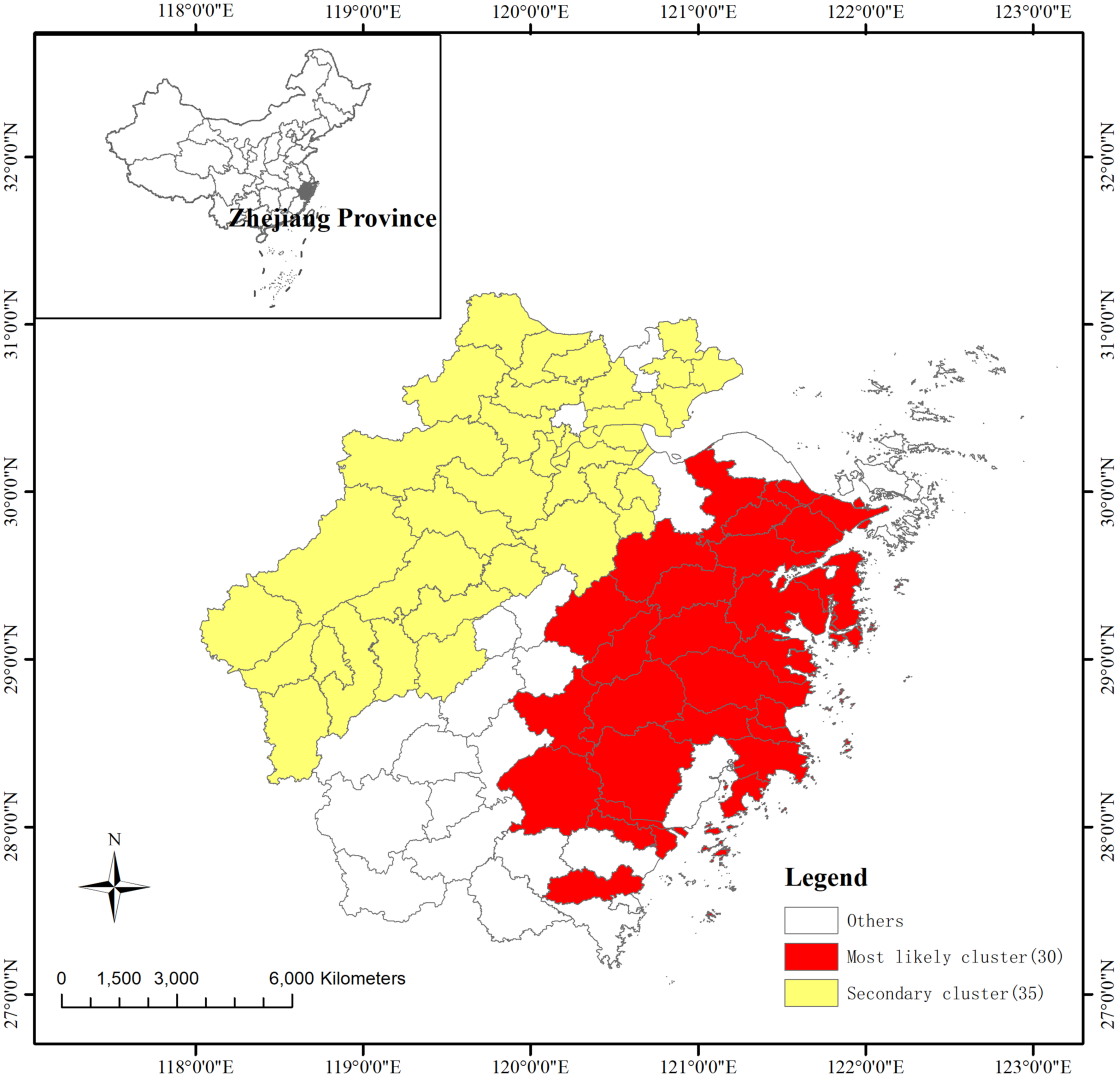
Spatiotemporal clusters of AHC cases in Zhejiang province from 2004 to 2023.

Using Kulldorff’s space–time scan statistic to detect spatiotemporal clusters across the study period, the result of the temporal scan showed that the most likely cluster was distributed in the southeast Zhejiang province and covered 30 counties (RR = 21.44, LRR = 5373.20, *p* < 0.001), with the time aggregation frame being 1st September 2010 to 30th September 2010 (Table 2). The secondary likely cluster located in the northwest regions of Zhejiang covered 35 counties (RR = 7.23, LRR = 1369.73, *p* < 0.001), which were recorded from 1st September 2010 to 30th September 2010 (Table 2; Figure 4).

**Table 2:** The cluster results of space-time scan for AHC cases in Zhejiang province from 2004 to 2023.

Cluster type	Time frame	Counties (n)	Radius (km)	Observed cases	Expected cases	LLR	RR	*p*-value
Most likely	2010/9/1–2010/9/30	30	1.40	12,740	79.26	53739.20	212.44	<0.001
Secondary	2010/9/1–2010/9/30	35	128.08	4,535	86.65	13695.73	57.23	<0.001

RR, relative risk; LLR, log likelihood ratio.

### Comparative demographic characteristics within and outside temporal and space–time clusters

The proportion of male AHC in high-risk counties was lower than that in low-risk counties with a statistically significant difference (*χ*^2^ = 5.525, *p* = 0.019). With regard to the difference in the age distribution of AHC, it was younger in cluster 1 than in the low-risk counties (Z = -2.355, *p* = 0.018), but it was older in cluster 2 (Z = 3.509, *p* < 0.001). The proportion of students in reported AHC cases in cluster 1 was lower than that in low-risk areas (*χ*^2^ = 169.241, *p* < 0.001), but it was higher in cluster 2 (*χ*^2^ = 99.703, *p* < 0.001). Additionally, AHC cases from cluster 1 had fewer days from illness onset to diagnosis with a median of 1 day (Z = -7.970, *p* < 0.001), while cluster 2 had longer days than low-risk areas (Z = 7.996, *p* < 0.001) (Table 3).

**Table 3:** Comparison of characteristics of AHC cases between high-risk counties and low-risk counties identified by Kulldorff’s spatiotemporal scan statistic in Zhejiang Province, 2004–2023.

Variables		High-risk counties		Low-risk counties
		Cluster 1	Cluster 2	
Gender	Male (%)	18,233 (57.83)*	7,770 (60.61)*	4,606 (59.29)
	Female (%)	13,298 (42.17)	5,050 (39.39)	3,162 (40.71)
Age	Median (Interquartile range)	23.00 (8–37)^#^	23.00 (10–39)*	23.00 (8–38)
Occupation	Students (%)	8,043 (25.51)^#^	4,122 (32.15)*	2,124 (27.34)
	Farmers (%)	4,615 (14.64)	2,841 (22.16)	1,542 (19.85)
	other (%)	18,873 (59.86)	5,857 (45.69)	4,102 (52.81)
Days from onset to diagnosis	Median (Interquartile range)	1.00 (0–2)^#^	1.00 (1–2)*	1.00 (1–2)

^#^Significant difference with lower value in high-risk counties compared with low-risk counties (*χ*^2^ test or Z test with *p* < 0.05).

*Significant difference with higher value in high-risk counties compared with low-risk counties (*χ*^2^ test or Z test with *p* < 0.05).

### Outbreak surveillance

**Figure 5:**
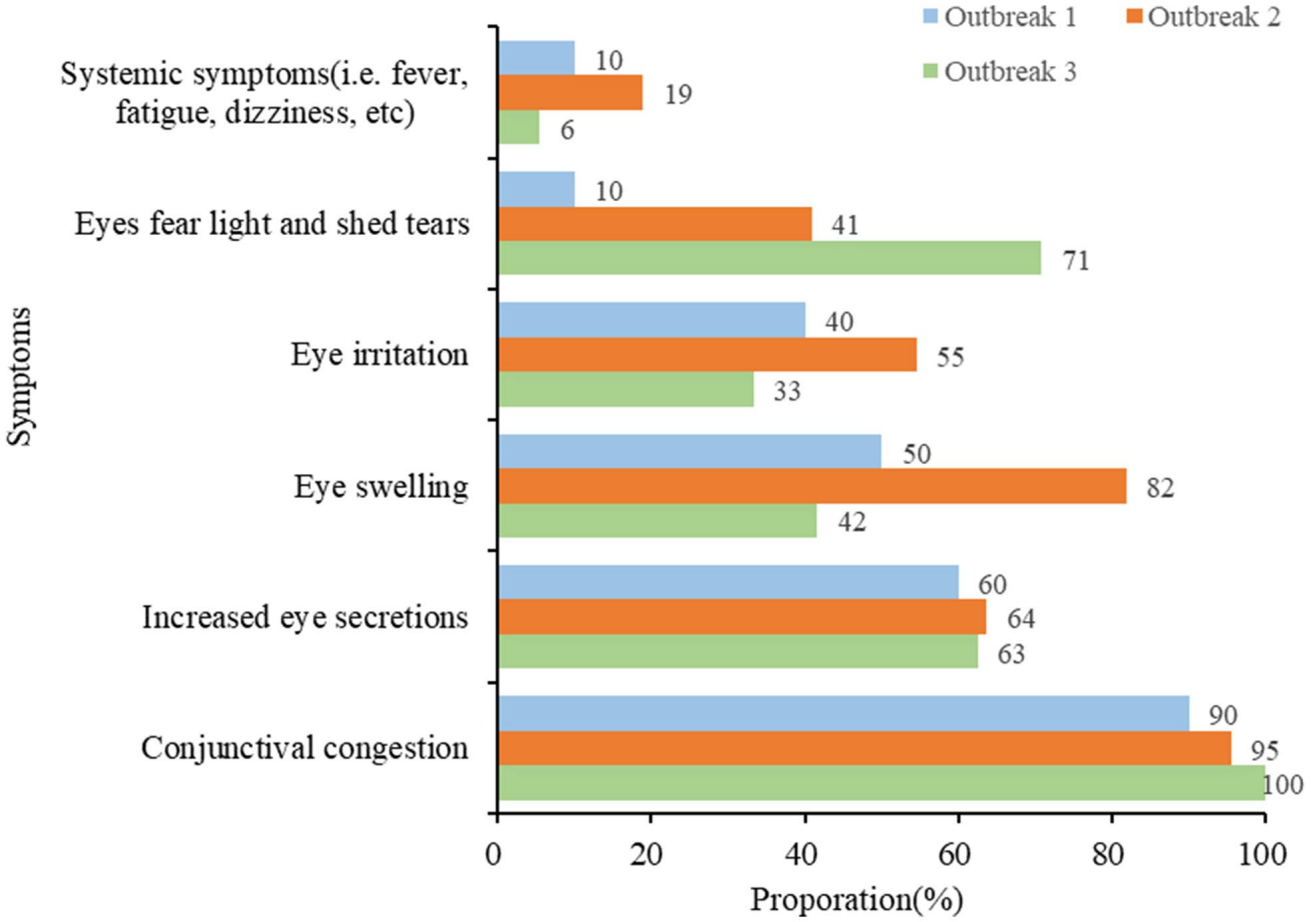
Distribution of clinical symptoms in three AHC outbreaks.

There were three outbreaks caused by AHC, all reported in September 2023 in primary schools. A total of 104 clinically confirmed cases have been reported, and no fatalities have been reported. In each outbreak, gender ratios of male vs. female cases were larger than 1(7:3; 6:5, 43:29, respectively), with an outbreak duration of 8.33 ± 2.42 days (Table 4). The symptoms of the infected students were mainly conjunctival congestion, increased eye secretions, eye swelling, eye pain, photophobia, and tearing, and the proportion of fever was relatively low (Figure 5).

**Table 4:** Basic information of three outbreaks reported in Zhejiang province from 2004 to 2023.

Name	Date of onset of the first case	Date of outbreak report	County	Place	Total number of students in the school	Number of clinically diagnosed cases	Gender ratio (male:female) of cases	Age range (years old)	Number of classes where cases occurred	Duration of outbreak (days)	Laboratory test results
Outbreak 1	2023/9/5	2023/9/11	Nanxun county	Primary school	2042	10	7:3	6–7	1class	6	Positive universal nucleic acid test for enterovirus
Outbreak 2	2023/9/12	2023/9/15	Kaihua county	Primary school	2058	22	6:5	6–7	5 classes	4	None
Outbreak 3	2023/9/14	2023/9/20	Fenghua county	Primary school	889	72	43:29	6–12	17 classes	15	Positive universal nucleic acid test for enterovirus

## Discussion

This study described the epidemiological characteristics and spatiotemporal patterns of AHC cases in Zhejiang province using the systematic dataset of monthly AHC during the 20-year study period from 2004 to 2023, which provided a good basis for understanding the epidemiological characteristics of the infectious disease. Incidence data suggested that the total number of AHC male cases was higher than female cases in this study, and the sex ratio of male to female was higher than the general population of Zhejiang Province in the same period. This finding is in concordance with the results reported in Malaysia and other studies (4, 26).

AHC occurs year-round, with a peak incidence in September, potentially due to climatic factors. A study by Zhang et al. (27) found a moderate positive correlation between AHC and monthly mean temperature, relative humidity, and precipitation. Other enteroviruses (EVs) have also been found to have a similar relationship with temperature factors, such as hand-foot-mouth disease (HFMD) (28, 29). However, the exact mechanism behind the relationship between temperature and EVs remains unclear. We speculate that the survival of the virus in the environment, virus replication, and human behavior are possible factors. First, virological studies showed that human EV was sensitive to temperature, and 20°C was the most suitable temperature for its survival (30, 31). Second, children tend to participate in more outdoor activities when the temperature is appropriate, so it may increase the spread of AHC by increasing close contact between individuals and between individuals and contaminated surfaces (30, 32). In the meantime, school holidays were significantly associated with lower incidence (27). In September, students return to school after summer vacation. The gathering of students has exacerbated the spread or outbreak of AHC (33), which may also explain why September has become the peak month for AHC’s popularity.

In summary, the epidemiological characteristics of AHC indicate that populations such as men, students, and farmers are more likely to be infected with AHC than other populations, which is consistent with the research results on the prevalence patterns of AHC in Chongqing (34) and Jinan (35). This phenomenon may be because (1) men tend to be hyperactive and engage in different types of occupational labor (36). (2) As a collective unit, the school has a crowding of individuals (37). Once there is a source of infection, students have more chances to be infected and even cause an outbreak. In addition, the evidence of school holidays was significantly associated with lower incidence, with IRRs of 0.91 (an 8.57% reduction) compared to the non-school holiday (27), which has been proven that student gatherings during school contribute to the occurrence of AHC. (3) AHC is easy to spread in densely populated areas with poor medical and health conditions. Farmers lack sufficient health knowledge and good hygienic habits and live in places with insufficient medical and health facilities. The infection source was not effectively controlled and isolated, which is an important cause of infection (38).

This study found that it is difficult to collect laboratory diagnostic results of AHC-infected individuals, and the vast majority of these cases are clinical diagnosis cases defined by clinical doctors based on the patient’s clinical symptoms, physical signs, and cytological examination. The lack of laboratory-confirmed results of cases may be because the symptoms of AHC infection include unique bulbar conjunctival bleeding follicular conjunctival reactions, and this infection has a short duration and self-limitation (39). Symptomatic treatment appears to be as effective as medical regimens to relieve symptoms. Therefore, doctors or patients are not motivated to retain samples for laboratory testing. To increase the proportion of pathogen detection in AHC cases, an active monitoring system should be established in the future to collect specimens from AHC patients and send them to the laboratory for testing instead of relying solely on existing passive monitoring systems.

Our study revealed that the incidence remained stable except for the years 2007, 2008, 2010, and 2011. Zhejiang had experienced the highest epidemic level so far in 2010. A similar situation of high AHC incidence also occurred in Guangxi, Guangdong, Chongqing, Shandong, and other regions in the year 2010 (34). In this study, we have found counties along the southeast coast of Zhejiang Province have higher incidence rates, which may be because coastal cities have more rainfall and migrant populations than inland cities (40). Results of multivariable analysis from other studies revealed that floods, city urbanization, and per capita play an important role in the occurrence of AHC. When a flood occurs, it pollutes water sources, worsens the living environment, and makes AHC easy to spread (41, 42). Additionally, urbanization and low per capita usually mean poorer sanitary conditions and heavy traffic, making pathogens spread rapidly (7). Compared with the pre-pandemic of COVID-19 in the year 2019 and the post-pandemic of COVID-19 in the year 2023, 2020–2022 had the minimum incidence of AHC incidence, which may be affected by non-intervention measures during the pandemic of COVID-19 (43).

The global autocorrelation analysis revealed that AHC cases in Zhejiang province were not randomly distributed, indicating spatial clustering. Further LISA analysis results demonstrated that the high-high clusters varied over time. These areas have abundant precipitation, which is conducive to the occurrence of AHC. Temporal and space–time cluster analysis identified two high-risk clusters, which were generally consistent with local spatial autocorrelation results but also exhibited some differences. The two likely clusters include a total of 65 counties, accounting for 73% of the total number of districts in the whole of Zhejiang province. This indicates that large-scale outbreaks may exist from 1st September to 30th September 2010. Unfortunately, the monitoring network has not received any information on the outbreak during the same period. Only three outbreaks in schools were reported in September 2023 (which was probably due to the government strengthening the monitoring and reporting requirements for school epidemics due to the 19th Asian Games held in Hangzhou from 23rd September to 8th October 2023); clinical symptoms, including conjunctival congestion, increased eye secretions, eye swelling, eye irritation, fear of light and shedding tears, are the main clinical manifestations, which align with prior India and Thailand studies (12, 33, 44). Results of spatial clustering and outbreak characteristics suggest that the intervention of health administrative departments plays an important role in the improvement of AHC monitoring work. To further improve the level of disease prevention and control in areas with high incidence rates of AHC, recommendations for various levels of health administrative departments and public health practitioners are listed as follows: (1) Increasing the laboratory tests of patients with related symptoms in hospitals to make a clear diagnosis and implementing preferential measures for the test costs; (2) Strengthening the awareness of disease monitoring in schools and carry out daily morning and afternoon examinations for students in epidemic seasons; (3) Expanding the population scope of health education on disease prevention and control; and (4) The communication mechanism for the guidance of schools and other collective units on reporting and outbreaks disposal measures should be unblocked to control the number of infections in the outbreak as soon as possible.

Despite our research providing significant new insights into the epidemiological characteristics of AHC in Zhejiang province, it is important to consider two limitations when interpreting our findings. First, there is an inherent bias due to the use of passive surveillance data, which only includes cases that sought medical care in hospitals and were subsequently reported to the local disease control and prevention center for analysis. The quality of the data might also be affected by the availability of diagnostic techniques and the varying experience levels of doctors across different hospitals. Second, we failed to obtain the etiological classification of AHC cases; all cases were clinically diagnosed.

## Conclusion

In summary, this study represents the first comprehensive and detailed analysis of the spatiotemporal dynamics of AHC in Zhejiang province from 2004 to 2023. Currently, no specific preventive measures are available for AHC. Transmission occurs mainly through eye-to-hand, object, and water. We recommend implementing public education for the population, improving their living environment, and enhancing accessibility to medical and health resources to prevent and control AHC outbreaks effectively. Our study demonstrates geographical distribution and seasonal variation in Zhejiang province, providing crucial evidence to health authorities and policymakers for optimizing the allocation of limited resources for disease prevention.

## Data Availability

The original contributions presented in the study are included in the article/Supplementary material. Further inquiries can be directed to the corresponding author.

## References

[1] ZHANG L ZHAO N SHA J WANG C JIN X AMER S. Virology and epidemiology analyses of global adenovirus-associated conjunctivitis outbreaks, 1953-2013. Epidemiol Infect. (2016) 144:1661–72. doi: 10.1017/S0950268815003246, PMID: 26732024 PMC9150610

[2] Zhang L Zhao N Huang X Jin X Geng X Chan TC. Molecular epidemiology of acute hemorrhagic conjunctivitis caused by coxsackie a type 24 variant in China, 2004-2014. Sci Rep. (2017) 7:45202. doi: 10.1038/srep45202, PMID: 28332617 PMC5362916

[3] Medina NH Haro-Muñoz E Pellini AC Machado BC Russo DH Timenetsky MD. Acute hemorrhagic conjunctivitis epidemic in São Paulo State, Brazil, 2011. Rev Panam Salud Publica. (2016) 39:137–141. doi: 10.1590/S1020-49892013000200007 27754516

[4] Liu J Zhang H. Epidemiological investigation and risk factor analysis of acute hemorrhagic conjunctivitis in Huangshi Port District. Huangshi City Comput Math Methods Med. (2022) 2022:3009589. doi: 10.1155/2022/3009589 35547568 PMC9085324

[5] Yoo B Kim MG Min AY Seo DW Kim SH Kim SH. Optimization of RT-PCR methods for enterovirus detection in groundwater. Heliyon. (2023) 9:e23028. doi: 10.1016/j.heliyon.2023.e23028, PMID: 38149210 PMC10750030

[6] Hu Tingting ZH Xiaojian D Xiaoxia H Xiaofang W Ying W. Epidemiological characteristics of acute hemorrhagic conjunctivitis in China, 2013–2020. Disease Surveillance, (2021). 36:5.

[7] Liu R Chen Y Liu H Huang X Zhou F. Epidemiological trends and sociodemographic factors associated with acute hemorrhagic conjunctivitis in mainland China from 2004 to 2018. Virol J. (2022) 19:34. doi: 10.1186/s12985-022-01758-6, PMID: 35232483 PMC8889670

[8] Devilliers MJ Ben W Barreau E da E M’Garrech M Bénichou J. Ocular manifestations of viral diseases. Rev Med Interne. (2021) 42:401–10. doi: 10.1016/j.revmed.2020.08.022, PMID: 33168354 PMC7646372

[9] Chatterjee S Quarcoopome C Apenteng AQC Apenteng A. Unusual type of epidemic conjunctivitis in Ghana. Br J Ophthalmol. (1970) 54:628–30. doi: 10.1136/bjo.54.9.628, PMID: 5458256 PMC1207974

[10] Baggen J Hurdiss DL Zocher G Mistry N Roberts RW Slager JJ. Role of enhanced receptor engagement in the evolution of a pandemic acute hemorrhagic conjunctivitis virus. Proc Natl Acad Sci USA. (2018) 115:397–402. doi: 10.1073/pnas.1713284115, PMID: 29284752 PMC5777043

[11] Oh MD Park S Choi Y Kim H Lee K Park W. Acute hemorrhagic conjunctivitis caused by coxsackievirus A24 variant, South Korea, 2002. Emerg Infect Dis. (2003) 9:1010–2. doi: 10.3201/eid0908.030190, PMID: 12967504 PMC3020616

[12] Chansaenroj J Vongpunsawad S Puenpa J Theamboonlers A Vuthitanachot V Chattakul P. Epidemic outbreak of acute haemorrhagic conjunctivitis caused by coxsackievirus A24 in Thailand, 2014. Epidemiol Infect. (2015) 143:3087–3093. doi: 10.1017/S0950268815000643, PMID: 25824006 PMC9151063

[13] Chang WKLK Foo TC Lam MW Chan CF. Acute haemorrhagic conjunctivitis in Hong Kong 1971-1975. Southeast Asian J Trop Med Public Health. (1977) 8:6. 196351

[14] National Health Commission of the People’s Republic of China. Statistical bulletin of China’s health development in 2022. (2023). Available at: http://www.nhc.gov.cn/guihuaxxs/s3585u/202309/6707c48f2a2b420fbfb739c393fcca92/files/8a3994e41d944f589d914c589a702592.pdf.

[15] Gao S Geng X Lu Q Wu S Shan Z Chang C. Epidemiological characteristics and spatio-temporal aggregation of severe fever with thrombocytopenia syndrome in Jinan City, China, 2018-2022. PLoS Negl Trop Dis. (2023) 17:e0011807. doi: 10.1371/journal.pntd.0011807, PMID: 38134002 PMC10745217

[16] Liang S Li Z Zhang N Wang X Qin Y Xie W. Epidemiological and spatiotemporal analysis of severe fever with thrombocytopenia syndrome in eastern China, 2011-2021. BMC Public Health. (2023) 23:508. doi: 10.1186/s12889-023-15379-3, PMID: 36927782 PMC10019416

[17] Zhejiang Provincial Bureau of Statistics. Natural geography of Zhejiang Province. (2024). Available at: https://tjj.zj.gov.cn/col/col1525489/index.html.

[18] National Health Commission of the People’s Republic of China. Diagnosis standard of acute hemorrhagic conjunctivitis. (2008). Available at: https://icdc.chinacdc.cn/zcfgybz/bz/202112/P020211202499583275686.pdf.

[19] Cliff ADOJ. Spatial processes: Models & Applications. London: Taylor & Francis (1981).

[20] Zhang WY Wang LY Liu YX Yin WW Hu WB Magalhaes RJS. Spatiotemporal transmission dynamics of hemorrhagic fever with renal syndrome in China, 2005-2012. PLoS Negl Trop Dis. (2014) 8:e3344. doi: 10.1371/journal.pntd.0003344 25412324 PMC4239011

[21] Yang S Liu X Gao Y Chen B Lu L Zheng W. Spatiotemporal dynamics of scrub typhus in Jiangxi Province, China, from 2006 to 2018. Int J Environ Res Public Health. (2021) 18:4599. doi: 10.3390/ijerph18094599, PMID: 33926106 PMC8123664

[22] Wu YC Qian Q Soares Magalhaes RJ Han ZH Hu WB Haque U. Spatiotemporal dynamics of scrub typhus transmission in mainland China, 2006-2014. PLoS Negl Trop Dis. (2016) 10:e0004875. doi: 10.1371/journal.pntd.0004875, PMID: 27479297 PMC4968795

[23] Wei Y Huang Y Luo L Xiao X Liu L Yang Z. Rapid increase of scrub typhus: an epidemiology and spatial-temporal cluster analysis in Guangzhou City, southern China, 2006-2012. PLoS One. (2014) 9:e101976. doi: 10.1371/journal.pone.0101976, PMID: 25006820 PMC4090214

[24] Qian L Wang Y Wei X Liu P Magalhaes RJS Qian Q. Epidemiological characteristics and spatiotemporal patterns of scrub typhus in Fujian province during 2012-2020. PLoS Negl Trop Dis. (2022) 16:e0010278. doi: 10.1371/journal.pntd.0010278, PMID: 36174105 PMC9553047

[25] Kulldorff M., SaTScan users guide. (2022).

[26] Ghazali O Chua KB Ng KP Hooi PS Pallansch MA Oberste MS. An outbreak of acute haemorrhagic conjunctivitis in Melaka, Malaysia. Singapore Med J. (2003) 44:511–516. 15024454

[27] Zhang L Jiang H Wang K Yuan Y Fu Q Jin X. Long-term effects of weather condition and air pollution on acute hemorrhagic conjunctivitis in China: a nationalwide surveillance study in China. Environ Res. (2021) 201:111616. doi: 10.1016/j.envres.2021.111616, PMID: 34233156

[28] Feng H Duan G Zhang R Zhang W. Time series analysis of hand-foot-mouth disease hospitalization in Zhengzhou: establishment of forecasting models using climate variables as predictors. PLoS One. (2014) 9:e87916. doi: 10.1371/journal.pone.0087916, PMID: 24498221 PMC3909295

[29] Wu H Wang H Wang Q Xin Q Lin H. The effect of meteorological factors on adolescent hand, foot, and mouth disease and associated effect modifiers. Glob Health Action. (2014) 7:24664. doi: 10.3402/gha.v7.24664, PMID: 25098727 PMC4124175

[30] Wang P Goggins WB Chan EY. Hand, foot and mouth disease in Hong Kong: a time-series analysis on its relationship with weather. PLoS One. (2016) 11:e0161006. doi: 10.1371/journal.pone.0161006, PMID: 27532865 PMC4988669

[31] Rzezutka A Cook N. Survival of human enteric viruses in the environment and food. FEMS Microbiol Rev. (2004) 28:441–53. doi: 10.1016/j.femsre.2004.02.001, PMID: 15374660

[32] Wang C Li X Zhang Y Xu Q Huang F Cao K. Spatiotemporal cluster patterns of hand, foot, and mouth disease at the county level in mainland China, 2008-2012. PLoS One. (2016) 11:e0147532. doi: 10.1371/journal.pone.0147532, PMID: 26809151 PMC4726594

[33] Boro P Gongo T Ori K Kamki Y Ete N Jini M. An outbreak of acute hemorrhagic conjunctivitis due to Coxsackievirus A24 in a residential school, Naharlagun, Arunachal Pradesh: July 2023. Indian J Med Microbiol. (2024) 48:100549. doi: 10.1016/j.ijmmb.2024.100549, PMID: 38395257

[34] Jing D Zhao H Ou R Zhu H Hu L Giri M. Epidemiological characteristics and spatiotemporal analysis of acute hemorrhagic conjunctivitis from 2004 to 2018 in Chongqing, China. Sci Rep. (2020) 10:9286. doi: 10.1038/s41598-020-66467-y, PMID: 32518362 PMC7283237

[35] Xu H Liu X. Analysis of epidemiological characteristics of acute hemorrhagic conjunctivitis in the city of Jinan from 2004-2011. J Pathogen Biology. (2014) 9:827–829.

[36] Xu X Zhao Y Xia S Zhang X. Investigation of multi-scale spatio-temporal pattern of oldest-old clusters in China on the basis of spatial scan statistics. PLoS One. (2019) 14:e0219695. doi: 10.1371/journal.pone.0219695, PMID: 31348778 PMC6660084

[37] Chang Z Zhang J Wang Z. Analysis on epidemic feature of acute hemorrhagic conjunctivitis from 2004 to 2007 in China. Chinese J Public Health Mangement. (2009):3.

[38] Yang J Qiao L Zou J Xiang Y Tian L Ren H. Epidemiological features of acute hemorrhagic conjunctivitis cases in Shi-fung city of Si-chuan province from 2006 to 2010. Int J Virol. (2011) 18:5.

[39] Sklar VE Patriarca PA Onorato IM Langford MP Clark SW Culbertson WW. Clinical findings and results of treatment in an outbreak of acute hemorrhagic conjunctivitis in southern Florida. Am J Ophthalmol. (1983) 95:45–54. doi: 10.1016/0002-9394(83)90332-X, PMID: 6849368

[40] Deng L Zhang L Fan X Sun T Fei K Ni L. Effects of rainfall intensity and slope gradient on runoff and sediment yield from hillslopes with weathered granite. Environ Sci Pollut Res Int. (2019) 26:32559–73. doi: 10.1007/s11356-019-06486-z, PMID: 31628640

[41] Ding G Li X Li X Zhang B Jiang B Li D. A time-trend ecological study for identifying flood-sensitive infectious diseases in Guangxi, China from 2005 to 2012. Environ Res. (2019) 176:108577. doi: 10.1016/j.envres.2019.108577, PMID: 31306984 PMC7094502

[42] Liu X Qiu S Liu Z Chen D Liu H Ding G. Effects of floods on the incidence of acute hemorrhagic conjunctivitis in Mengshan, China, from 2005 to 2012. Am J Trop Med Hyg. (2020) 102:1263–8. doi: 10.4269/ajtmh.19-0164, PMID: 32228794 PMC7253130

[43] Zhang L Guo X Jiang H Zhao N Cheng W Xu W. Decreased incidence of acute hemorrhagic conjunctivitis associated with enhanced public health intervention during the COVID-19 epidemic in China, 2020. Arch Virol. (2022) 167:577–81. doi: 10.1007/s00705-021-05282-w, PMID: 35039974 PMC8763440

[44] Shukla D Kumar A Srivastava S Dhole TN. Molecular identification and phylogenetic study of coxsackievirus A24 variant isolated from an outbreak of acute hemorrhagic conjunctivitis in India in 2010. Arch Virol. (2012) 158:679–84. doi: 10.1007/s00705-012-1520-7, PMID: 23124888

