# Supplementary material for: Epidemiology and spatiotemporal analysis of acute hemorrhagic conjunctivitis in Zhejiang province, China (2004–2023)
Source: Front Public Health. 2025 Feb 3;13:1509495. doi: 10.3389/fpubh.2025.1509495 (PMC11833792; doi:10.3389/fpubh.2025.1509495)
Supplement: Supplementary file 1 [file Supplementary_file_1.docx]

**S1. Diagnostic criteria for AHC cases (translated from reference 18 in the article)**

4 Diagnostic criteria

4.1 Epidemiological history

4.1.1 Travel history

A travel history to a community, family, or workplace where an acute hemorrhagic conjunctivitis case occurred within 6 days prior to the onset of the disease.

4.1.2 Contact History

There is a history of direct or indirect contact with patients with acute hemorrhagic conjunctivitis within 6 days before the onset of the disease.

4.2 Clinical manifestations

4.2.1 Symptoms

The incubation period is short, generally ranging from 12 hours to 48 hours, and can last up to 6 days; The onset is sudden, with obvious symptoms, and both eyes are affected successively or simultaneously; There are severe symptoms of foreign body sensation, eye redness, eye pain, photophobia, tearing and other irritants; Early secretions are aqueous, with some being light red in color, and then mucous (see Appendix A.3).

4.2.2 Physical Signs

Eyelid redness and swelling, high congestion in the eyelid and conjunctiva, often accompanied by subconjunctival and patchy bleeding. Early corneal epithelial punctate peeling, after fluorescein staining and slit lamp examination, diffuse scattered small punctate staining of the cornea can be seen, accompanied by preauricular lymph node enlargement, etc. (see Appendix A.3).

4.3 Laboratory Testing

4.3.1 Cytological examination

Conjunctival cytology examination showed mainly monocyte and lymphocyte reactions.

4.3.2 Virus isolation and identification

The virus was isolated by conjunctival swab application or conjunctival scraping culture, and identified as EV70 or CA24v or adenovirus using micro neutralization experiments or PCR methods.

4.3.3 Antigen testing

Indirect immunofluorescence technology was used to detect EV70 or CA24v antigens or adenovirus antigens in conjunctival cell smears or cell culture smears.

4.3.4 Serological examination

Bipolar serological examination shows that the titer of anti-EV70, anti-CA24v, or adenovirus antibodies in the patient's recovery phase serum is ≥ 4 times higher than that in the acute phase serum antibody.

4.3.5 Fluorescence quantitative RT-PCR nucleic acid detection

Fluorescent quantitative RT-PCR detection for specific nucleic acid positivity of EV70, CA24v, or adenovirus.

5 Diagnostic Principles

The principle of comprehensive judgment should be followed. Based on epidemiological history, clinical symptoms, and signs, combined with conjunctival cytology examination, a clinical diagnosis is made. A clinical diagnosis is made by combining pathogen testing, serological testing, or fluorescence quantitative RT-PCR nucleic acid testing to confirm the diagnosis.

6 Diagnosis

6.1 Suspected Cases

Those who meet both criteria 4.1 and 4.2 simultaneously.

6.2 Clinical Diagnosis Cases

Those who meet the requirements of items 4.1, 4.2, and 4.3.1 simultaneously.

6.3 Confirmed Cases

Simultaneously comply with 4.1, 4.2, and any one of 4.3.2, 4.3.3, 4.3.4, 4.3.5.
